# Beta3Gn-T7 Is a Keratan Sulfate β1,3 *N*-Acetylglucosaminyltransferase in the Adult Brain

**DOI:** 10.3389/fnana.2022.813841

**Published:** 2022-02-09

**Authors:** Yoshiko Takeda-Uchimura, Kazuchika Nishitsuji, Midori Ikezaki, Tomoya O. Akama, Yoshito Ihara, Fabrice Allain, Kenji Uchimura

**Affiliations:** ^1^Univ. Lille, CNRS, UMR 8576 – UGSF – Unité de Glycobiologie Structurale et Fonctionnelle, Lille, France; ^2^Department of Biochemistry, Wakayama Medical University, Wakayama, Japan; ^3^Department of Pharmacology, Kansai Medical University, Osaka, Japan

**Keywords:** keratan sulfate (KS), *B3gnt7*, *N*-acetylglucosaminyltransferase, oligodendrocyte, adult brain

## Abstract

Keratan sulfate (KS) glycan is covalently attached to a core protein of proteoglycans. KS is abundant in neuropils and presents densely in close proximity to the perineuronal region of the perineuronal net-positive neurons in the adult brain under physiological conditions. We previously showed that the synthesis of KS positive for the R-10G antibody in the adult brain is mediated by GlcNAc-6-sulfotransferase 3 (GlcNAc6ST3; encoded by *Chst5*). Deficiency in both GlcNAc6ST3 and GlcNAc6ST1, encoded by *Chst2*, completely abolished KS. Protein-tyrosine phosphatase receptor type z1 (Ptprz1)/phosphacan was identified as a KS scaffold. KS requires the extension of GlcNAc by β1,3 *N*-acetylglucosaminyltransferase (Beta3Gn-T). Members of the Beta3Gn-T family involved in the synthesis of adult brain KS have not been identified. In this study, we show by a method of gene targeting that Beta3Gn-T7, encoded by *B3gnt7*, is a major Beta3Gn-T for the synthesis of KS in neuropils and the perineuronal region in the adult brain. Intriguingly, the *B3gnt7* gene is selectively expressed in oligodendrocyte precursor cells (OPCs) and oligodendrocytes similar to that of GlcNAc6ST3. These results indicate that Beta3Gn-T7 in oligodendrocyte lineage cells may play a role in the formation of neuropils and perineuronal nets in the adult brain through the synthesis of R-10G-positive KS-modified proteoglycan.

## Introduction

Interstitial extracellular components such as proteoglycans, hyaluronan, and tenascins account for approximately 20% of the total volume of the adult brain (Nicholson and Rice, 1986). Extracellular proteoglycans and their sulfated glycosaminoglycan (GAG) components restrict synaptic plasticity and regulate axonal regrowth/sprouting (Silver et al., 2014; Fawcett, 2015). GAGs also implicate myeloid cells, such as microglia, in their functions in neuroinflammation (Gaudet and Popovich, 2014; O’callaghan et al., 2015; Zhang et al., 2017; Dyck et al., 2018). Keratan sulfate (KS) is a class of GAGs extended from *N*-linked, *O*-GalNAc linked, or *O*-mannose-linked oligosaccharides of the core protein. KS building blocks consist of repeating N-acetyllactosamine (LacNAc) disaccharides of galactose (Gal) and *N*-acetylglucosamine (GlcNAc) (Uchimura, 2015; Caterson and Melrose, 2018). It is known that the vast majority of GlcNAc residues are C-6 sulfated and that KS non-reducing termini are modified with different types of monosaccharides, such as sialic acids, which have the potential to interact with Siglecs (Zhang et al., 2017; Bull et al., 2021; Gonzalez-Gil and Schnaar, 2021; Lunemann et al., 2021; Smith and Bertozzi, 2021). KS glycans immunopositive for R-10G (Kawabe et al., 2013) and 5D4 (Caterson et al., 1983) monoclonal antibodies are expressed in the developing brains of humans, rats, and mice (Miller et al., 1997; Hoshino et al., 2014; Takeda-Uchimura et al., 2015; Melrose, 2019; Sarnat, 2019). These antibody epitopes are also expressed in the adult brains of humans and rats. However, negligible levels of the 5D4 epitope have been reported in the physiological processes of the adult mouse brain. Two species of KS proteoglycans are present in the brain, namely, neuropil R-10G-positive KS proteoglycan and microglial 5D4-positive KS proteoglycan. (i) *Neuropil R-10G-positive KS proteoglycan*, which is mostly assigned to the protein tyrosine phosphatase receptor type z1 (Ptprz1, also known as PTPRZ, PTP-ζ, or RPTPβ)/phosphacan, is expressed in both developing and adult brains (Takeda-Uchimura et al., 2015; Narentuya et al., 2019). In humans and rats, this species is also positive for 5D4 in both the developing and adult stages (Lindahl et al., 1996; Miller et al., 1997; Jones and Tuszynski, 2002). In mice, this species is positive for 5D4 during the developing period but not in adulthood (Hoshino et al., 2014; Narentuya et al., 2019). (ii) *Microglial 5D4-positive KS proteoglycan* is expressed in adult brains of patients and disease models of rats and mice. The expression of this molecule is induced in activated microglia under pathological conditions (Jones and Tuszynski, 2002; Fan et al., 2007; Xu et al., 2008; Hirano et al., 2013; Foyez et al., 2015; Zhang et al., 2017; Ohgomori and Jinno, 2020) and relies on GlcNAc-6-sulfotransferase (GlcNAc6ST) 1 (Uchimura et al., 1998; Zhang et al., 2017). In rats, a subset of resting microglia also shows an expression of the 5D4 KS proteoglycan (Bertolotto et al., 1993; Jander and Stoll, 1996; Jander et al., 2000; Jones and Tuszynski, 2002). The length of KS in this species is thought to be rather short. The non-reducing termini are modified with sialic acids (Zhang et al., 2017).

R-10G-positive KS in the neuropils and proximities of perineuronal nets (PNNs) of neurons largely depends on GlcNAc6ST3. GlcNAc6ST3 is selectively expressed in oligodendrocyte precursor cells (OPCs) and newly formed oligodendrocytes in the adult brain (Narentuya et al., 2019). It has been proposed that R-10G-positive KS, which is covalently linked to Ptprz1/phosphacan in neuropils and pericellular spaces of neurons, may be derived from the oligodendrocyte lineage cells. The extension of LacNAc disaccharide units of KS is mediated by Golgi-resident β1,3 *N*-acetylglucosaminyltransferase (Beta3Gn-T). Based on their enzymatic activities and specificities *in vitro*, eight members belong to the Beta3Gn-T family (Schjoldager et al., 2020). Although GlcNAc6STs for the brain KS have been extensively studied, Beta3Gn-T responsible for the synthesis of R-10G-positive KS in the adult brain has not been identified. In this study, we show that Beta3Gn-T7, also known as B3GNT7 encoded by *B3gnt7*, is essential for the synthesis of R-10G-positive KS in the adult brain and that cell types that selectively express Beta3Gn-T7 are OPCs and newly formed oligodendrocytes similar to those for GlcNAc6ST3. These results indicate that Beta3Gn-T7 in the oligodendrocyte lineage cells is a major KS enzyme in the adult brain. Beta3Gn-T7 may mediate the synthesis of R-10G-positive KS-modified Ptprz1/phosphacan, a PNN component, in concerted actions with GlcNAc6ST3.

## Materials and Methods

### Antibodies and Reagents

The following materials were obtained commercially from the indicated sources: The R-10G anti-GlcNAc-6-sulfated KS antibody (Kawabe et al., 2013; Nakao et al., 2017; Wu et al., 2019) was purchased from Cosmo Bio (Tokyo, Japan); mouse anti-β-actin antibody was purchased from Sigma (St. Louis, MO, United States); Cy*™*3-conjugated goat anti-mouse IgG1, horseradish peroxidase (HRP)-conjugated goat anti-mouse IgG1, and HRP-conjugated goat anti-mouse IgG2a were obtained from Jackson ImmunoResearch Laboratories (West Grove, PA, United States); and NeuroTrace*™* Fluorescent Nissl stain was purchased from Thermo Fisher Scientific (Waltham, MA, United States).

### Mice

GlcNAc6ST1-knockout (KO) mice (Uchimura et al., 2004, 2005) and GlcNAc6ST3-KO mice (Hayashida et al., 2006) were maintained on a C57BL/6J genetic background. GlcNAc6ST1 and GlcNAc6ST3 double-deficient knockout (DKO) mice were generated as described previously (Narentuya et al., 2019). B3gnt7-KO mice were previously generated by deleting exon 2 of the *B3gnt7* gene using recombination-mediated genetic engineering (Littlechild et al., 2018). B3gnt7-KO mice show a phenotype in the corneal organization but no gross abnormalities. Genotyping primers for B3gnt7-KO mice were used: B3gnt7KO_1stlox_upper: 5′-TGGACAGTGGTCTCTTTTCCTGG-3′, B3gnt7KO_1stlox _lower: 5′-AAGCACTGTGTATTCAGCTACTGG-3′, and B3gnt 7KO_2ndlox_lower: 5′-GTCTACTTCAATGCTTTCCGAAGG-3′. The B3gnt7KO_1stlox_upper and B3gnt7KO_1stlox_lower set amplified the sequence of the wild-type (WT) mouse *B3gnt7* allele and yielded a PCR product of 167 bp. The B3gnt7KO_1stlox_upper and B3gnt7KO_2ndlox_lower set amplified the sequence of the *B3gnt7* KO allele and yielded a PCR product of 110 bp. Male and female mice of all genotypes at 2 to 4-month-old were used for the experiments. All mice were maintained under controlled specific pathogen-free environmental conditions and provided with standard nourishment and water in the animal facilities of the institutions of the authors. All experiments were approved by the Animal Research Committee of the institutions of the authors and conducted according to the guidelines of the institutions of the authors.

### Mouse Tissues

Mice were anesthetized and transcardially perfused with phosphate-buffered saline (PBS). The brains were dissected and divided into sagittal parts. Regional parts of hemi-brains, namely, cerebral cortex, hippocampus, cerebellum, olfactory bulb, brainstem, and thalamus, were separated on ice, snap-frozen, and stored at −80°C for biochemical analysis. Hemi-brains for frozen sectioning were post-fixed overnight in phosphate buffer (PB) containing 4% paraformaldehyde, equilibrated into 30% sucrose in PBS, and then embedded in Tissue-Tek^®^ (O.C.T. compounds; Sakura, Torrance, CA, United States).

### Fractionation of Brain Samples

Snap-frozen brain samples (∼20 mg) were homogenized with a Dounce homogenizer in 600 μL (30 volumes of the tissue weight) of ice-cold Tris-buffered saline (TBS) containing 1% Triton X-100 (w/v) and cOmplete*™* protease inhibitor cocktail (Roche, Basel, Switzerland). The homogenized samples were placed on ice for 30 min, followed by centrifugation at 10,000*g* for 15 min at 4°C. The supernatants were heated for 10 min at 95°C, after which they were centrifuged at 10,000*g* for 3 min at 4°C. Supernatants were collected and used as the 1% Triton-soluble fraction. Protein concentration was measured using the Bradford method.

### Immunoblot

Twelve micrograms of proteins were separated using 5–20% gradient polyacrylamide gels (SuperSep, WAKO, Osaka, Japan) and blotted onto polyvinylidene difluoride membranes (Immobilon-P, Merck, Darmstadt, Germany). The membrane was blocked with protein-free EzBlock Chemi (ATTO, Tokyo, Japan) for 1 h at room temperature and then incubated with the R-10G anti-KS antibody (1:300 dilution) or mouse IgG control antibody (Vector Laboratories, Burlingame, CA, United States) at room temperature for 1 h. Membranes were then washed with TBS containing 0.1% Tween-20 (TBS-T) and incubated for 30 min at room temperature with HRP-conjugated goat anti-mouse IgG1 secondary antibody (1:25,000 dilution). Bound antibodies were detected using the ImmunoStar LD chemiluminescent reagent (WAKO) and a Lumino Graph image analyzer (ATTO). Densitometric analysis of immunoreactive bands was performed using ImageJ software^1^ (National Institutes of Health, Bethesda, MD, United States).

### Immunohistochemistry and Confocal Microscopy

Frozen brain tissues were cut into 10-μm-thick sections on a cryostat and collected on MAS-coated glass slides (SF17293; Matsunami, Osaka, Japan). Sections were air-dried for 30 min, rinsed with PBS to remove O.C.T. compounds, and then blocked in PBS containing 5% normal goat serum (Vector Laboratories) and 0.3% Triton-X 100 for 1 h at room temperature. Sections were incubated with R-10G (1:100 dilution) in PBS containing 0.03% Triton-X 100 at 4°C overnight. Sections were washed with PBS and incubated with Cy*™*3-anti-mouse IgG1 (1:400 dilution) for 30 min at room temperature. After washing with PBS, sections were incubated with NeuroTrace*™* 435/455 blue fluorescent Nissl stain to visualize the neurons. The stained sections were mounted in FluorSave*™* Reagent (Merck, Darmstadt, Germany). Signals were visualized and captured using a confocal microscope (A1Rsi, Nikon, Tokyo, Japan) at the same exposure settings for each antibody. The images were analyzed using the NIS-Elements Analysis software (Nikon).

### Gene Expression Patterns in Adult Brain Cells

Data pertinent to the *B3gnt7* gene were mined from a published RNA sequencing (RNA-Seq) analysis of purified neurons, OPCs, newly formed oligodendrocytes, myelinating oligodendrocytes, astrocytes, microglia, and endothelial cells from adult mouse brains (Zhang et al., 2014). Comparison of their transcription profiles in various cell types of the brain was performed using an RNA-Seq transcriptome platform^2^. The value of fragments per kilobase of transcript sequence per million mapped fragments (FPKM) of ∼0.04 was determined as a threshold for minimum gene expression (Zhang et al., 2014). The RNA-Seq dataset of the cell-types purified from mouse cerebral cortices was obtained from the National Center for Biotechnology Information Gene Expression Omnibus (Accession number GSE52564). FPKM values in the dataset were obtained and analyzed. Experimental conditions for grouping the purified populations into major cell-type classifications are comparable to the experiments performed in this study.

### Real-Time Quantitative Reverse Transcription-Polymerase Chain Reaction

Total RNA was extracted from frozen mouse cerebral cortices with the use of the RNeasy Lipid Tissue Mini Kit (Qiagen, Venlo, Netherlands) according to the protocol of the manufacturer. Real-time quantitative reverse transcription-PCR (RT-qPCR) was performed with the CFX96 Touch Real-time System (Bio-Rad Laboratories, Hercules, CA, United States) and iTaq Universal SYBR Green One-Step Kit (Bio-Rad Laboratories). Total RNA extracts (0.1 mg) were reverse-transcribed at 50°C for 10 min and then at 95°C for 1 min. PCRs were run for 40 cycles at 95°C for 10 s and 58°C for 30 s (*B3gnt7* and *B3gnt2*), at 95°C for 10 s and 56°C for 30 s (*B3gnt8* and *Gapdh*), at 95°C for 10 s and 54°C for 30 s (*B3gnt4* and *B3gnt9*), at 95°C for 10 s and 53°C for 30 s (*B3gnt3*), and at 95°C for 10 s and 52°C for 30 s (*B3gnt5* and *B3gnt6*). All RT-qPCR experiments were carried out in triplicate. The relative expression levels of each mRNA were calculated with the comparative ΔΔCt method and Bio-Rad CFX Manager version 3.1 (Bio-Rad Laboratories), normalizing to the level of *Gapdh*. Primer sequences were as follows: *B3gnt2*, forward: 5′-TCTGGTCTCAGTTGCAAAGTCCTAA-3′, reverse: 5′-GGCTACCTGCTCATGAAGGCTAA-3′ (Foyez et al., 2015); *B3gnt3*, forward: 5′-ATACGGCGACATTCTCCAG-3′, reverse: 5′-AAAGGACCTGCTTAAGCGT-3′; *B3gnt4*, forward: 5′-CTACCCACCTTATGCAGGA-3′, reverse: 5′-AAGATGCCT CACAGTAGCC-3′; *B3gnt5*, forward: 5′-ATGACTAACTGAA ACGTGGT-3′, reverse: 5′-GGTCTACCTCAACTTTCATCC-3′; *B3gnt6*, forward: 5′-TCTCAAACGCACAAGATGG-3′, reverse: 5′-CACACCCACTAGAAAGAAAGC-3′; *B3gnt7*, forward: 5′-T CCCAGCCGTCATGTATGGTAA-3′, reverse: 5′-ATGGTGG AGTTGCCGAGCTAA-3′ (Foyez et al., 2015); *B3gnt8*, forward: 5′-GCTCTGATAAGGATGTACCC-3′, reverse: 5′-GCAAAGT GTCCTGGTTCTG-3′; *B3gnt9*, forward: 5′-CTGAACTGACA GTTTCAGGG-3′, reverse: 5′-CATCCACTCTGTCGACCTC-3′; and *Gapdh*, forward: 5′-ACTCTTCCACCTTCGATGC-3′, reverse: 5′-CCGTATTCATTGTCATACCAGG-3′.

### Statistical Analysis

All data are presented as means ± SD. The values were analyzed by an ordinary one-way ANOVA with Tukey’s range test or unpaired *t*-test using Prism software (GraphPad Software, La Jolla, CA, United States). Differences were regarded as significant for *P* < 0.05.

## Results and Discussion

### Beta3Gn-T7 Is a Major β1,3 *N*-Acetylglucosaminyltransferase for R-10G-Positive Keratan Sulfate in the Adult Mouse Brain

**FIGURE 1:**
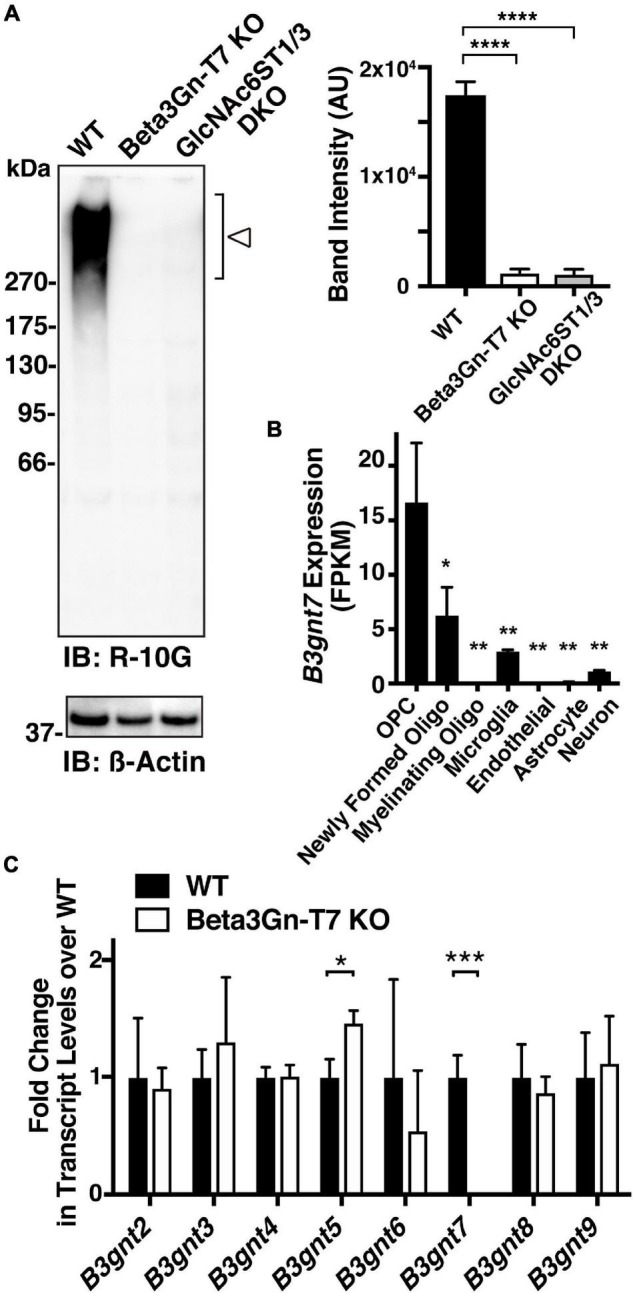
R-10G anti-keratan sulfate-staining signals in the neuropils and pericellular regions are negligible in the adult brain of Beta3Gn-T7 KO mice. Brain sections from adult WT and Beta3Gn-T7 KO mice were immunostained with R-10G (*red*) followed by NeuroTrace Nissl staining (*blue*). Representative confocal microscope images in layer 2/3 of the visual cortex are shown (*n* = 3 for WT and KO mice). Dense R-10G staining signals in the pericellular spaces (*arrowheads*) were deficient in Beta3Gn-T7 KO mice (*arrows*). Diffuse R-10G staining signals in intercellular spaces (*asterisks*) were deficient in Beta3Gn-T7 KO mice (*double asterisks*). Digital images were captured in the same settings for each staining. Scale bar: 10 μm.

Beta3Gn-T7, a member of the Beta3Gn-T family, was reported to have *N*-acetylglucosaminyltransferase activity toward GlcNAc-6-sulfated LacNAc-containing oligosaccharides *in vitro* (Seko and Yamashita, 2004) and in cultured cells (Kitayama et al., 2007). We wanted to determine if Beta3Gn-T7 was responsible for the R-10G-positive KS *in vivo*. We employed Beta3Gn-T7 gene KO mice (Littlechild et al., 2018). As we reported previously (Narentuya et al., 2019), R-10G staining signals in the neuropils and PNN proximities were seen within the whole area of the cerebral cortices. Surprisingly, diffuse R-10G signals in neuropils and dense pericellular signals could not be detected in the whole area of the adult brain, including the visual cortex, in Beta3Gn-T7 KO mice (Figure 1). Although the eight Beta3Gn-T members are expressed in the brain, deficiency in Beta3Gn-T7 resulted in a complete lack of the R-10G signals. This result indicated that Beta3Gn-T7 is a major Beta3Gn-T required for the synthesis of R-10G-positive KS in the adult brain. Under physiological conditions, the contributions of other members of the Beta3Gn-T family to the synthesis may be little or almost none.

### The R-10G-Immunoreactive Keratan Sulfate-Modified Proteins With a Size of >270 kDa Are Deficient in the Adult Beta3Gn-T7 Knockout Mouse Brain

**FIGURE 2:**
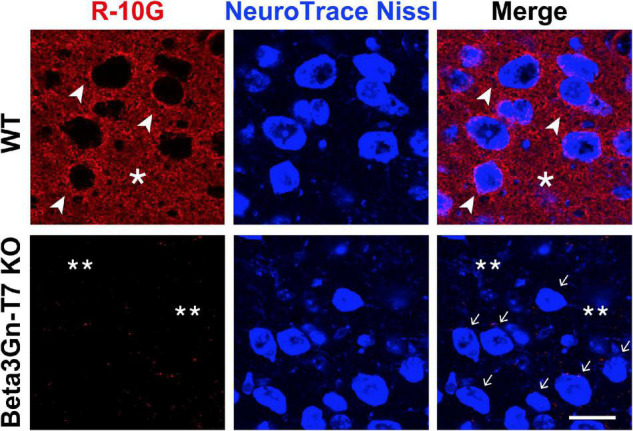
Beta3Gn-T7 is a major brain keratan sulfate β1,3 *N*-acetylglucosaminyltransferase in the cerebral cortex of adult mice. **(A)** Expression of the R-10G keratan sulfate epitope in the 1% Triton-soluble fractions prepared from the cerebral cortices of adult WT, Beta3Gn-T7 KO, and GlcNAc6ST1 and GlcNAc6ST3 double-deficient knockout (DKO) mice. GlcNAc6ST1 and GlcNAc6ST3 DKO were used as a negative control, since we previously showed that the R-10G epitope in the cerebral cortex was completely abolished in adult DKO mice (Narentuya et al., 2019). The band intensities with molecular weights of >270 kDa in the immunoblotting (*open arrowhead*) were measured by a densitometric quantitative analysis and shown in the bar graph at the right. The number of brain tissues used was three for each genotype. β-actin was used as a loading control. Data are presented as means ± SD, ****P < 0.0001. **(B)** RNA-Seq transcriptome results of the *B3gnt7* gene in indicated cell types are shown. Cells were prepared from the mouse cerebral cortices (Zhang et al., 2014). OPC, oligodendrocyte precursor cells. Data are presented as means ± SD, *P < 0.05 vs. OPC, **P < 0.01 vs. OPC. **(C)** Total RNA from the cerebral cortices of adult WT and Beta3Gn-T7 KO mice were prepared and tested. mRNA levels of eight members of the Beta3Gn-T family were determined by real-time quantitative PCR. The number of brain tissues used was three for each genotype. Data are presented as means ± SD, *P < 0.05, ***P < 0.001.

**FIGURE 3:**
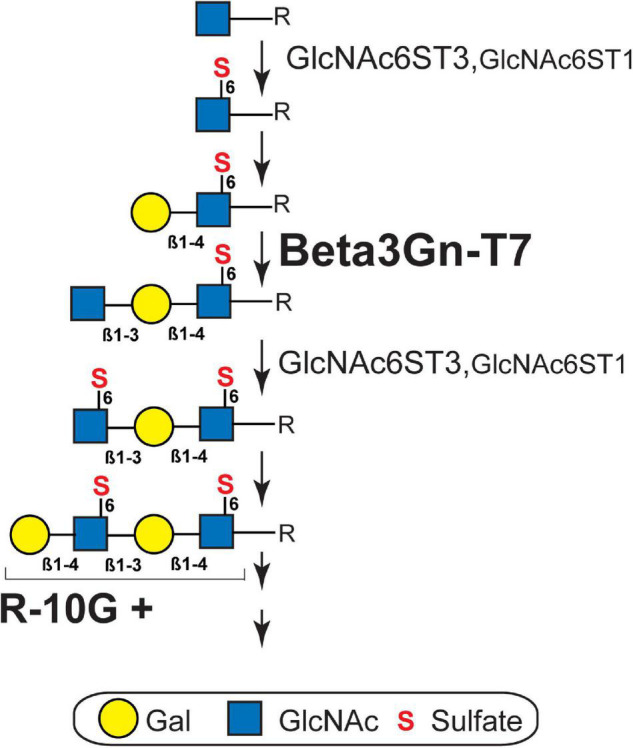
Biosynthesis pathway of brain keratan sulfate. R-10G-positive (R-10G +) brain keratan sulfate (KS) is extended from the variable underlying the core glycans (R). The sequence of biosynthesis is C-6 sulfation (S) of *N*-acetylglucosamine (GlcNAc) residue exposed at the non-reducing terminal by GlcNAc6ST3 or GlcNAc6ST1 (Narentuya et al., 2019), transfer of galactose (Gal) by a β1,4-galactosyltransferase, and then the transfer of GlcNAc by β1,3 *N*-acetylglucosaminyltransferase 7 (Beta3Gn-T7; shown in this study). GlcNAc6ST3 is a major KS GlcNAc6ST in the adult brain (Narentuya et al., 2019) and its contribution to KS synthesis in the developing brain is partially complemented by GlcNAc6ST1 (Takeda-Uchimura et al., 2015). β1,4-galactosyltransferase for KS synthesis in adult brains has not yet been identified. The KS structure extending from the R-10G + epitope can be additionally sulfated at some Gal residues by KSGal6ST (Hoshino et al., 2014). The length and degree of sulfation of cerebral KS vary during brain development, maturation, and physiopathology.

Ptprz1/phosphacan carries both chondroitin sulfate (CS) and KS (Rauch et al., 1991; Faissner et al., 1994; Garwood et al., 1999) and is distributed diffusely within the extracellular space of the brain (Deepa et al., 2006). We molecularly identified R-10G-immunoprecipitated proteins as isoforms of Ptprz1/phosphacan in the mouse cerebral cortex (Narentuya et al., 2019). In immunoblots of lysates prepared from adult cerebral cortices with the R-10G antibody, we detected a smear >270 kDa band in WT mice (Figure 2A). R-10G immunoreactivity was abolished in the lysate of Beta3Gn-T7 KO brains, as was also observed in GlcNAc6ST1 and GlcNAc6ST3 DKO mice (Narentuya et al., 2019; Figure 2A). Together with the results of R-10G immunohistochemistry, we concluded that Beta3Gn-T7 is a major Beta3Gn-T required for R-10G-positive KS present in neuropils and in the vicinity of the subsets of neurons in the adult brain. We then asked which cell types expressed the Beta3Gn-T7. Interestingly, the mRNA of the Beta3Gn-T7 gene, *B3gnt7*, was extensively expressed in OPCs (16.6 FPKM) and newly formed oligodendrocytes (6.2 FPKM) (Figure 2B). These results indicate that a major source of the R-10G-positive KS Ptprz1/phosphacan in neuropils and in the vicinity of the subsets of neurons may be the oligodendrocyte lineage cells in the adult brain. The level of expression in microglia (2.9 FPKM) could be attributable to the synthesis of other epitopes of KS or KS-related glycans that are not R-10G-positive. Under pathological conditions, microglial KS or KS-related glycan epitopes recognized by 5D4 are newly induced on the cell surface. These glycans in microglia are synthesized by GlcNAc6ST1 (Foyez et al., 2015; Zhang et al., 2017). We previously found that the Beta3Gn-T7 gene is upregulated in a pathological set of neurodegeneration along with GlcNAc6ST1 (Foyez et al., 2015). It is not clear whether Beta3Gn-T7 is also involved in the synthesis of 5D4-positive KS or KS-related glycans induced in activated microglia. Analysis of the Beta3Gn-T7 KO brain under neurodegenerative conditions may address this issue. *In situ* hybridization analysis for the localization of Beta3Gn-T7 mRNA may consolidate the cell-type-specific expression of the Beta3Gn-T7 gene. We then asked if the deficiency in Beta3Gn-T7 could downregulate other Beta3Gn-T family members. We measured the mRNA expression levels of all eight Beta3Gn-Ts (Beta3Gn-T2-9). The undetectable level of mRNA expression of Beta3Gn-T7 was corroborated in the Beta3Gn-T7 KO cerebral cortex (Figure 2C). The mRNA expression levels of Beta3Gn-T2, 3, 4, 6, 8, and 9 in the KO cerebral cortex were comparable with those of the WT control. Interestingly, the level of mRNA expression of Beta3Gn-T5, an enzyme specific to ceramides (Togayachi et al., 2001), resulted in an increase (1.5-fold) in the Beta3Gn-T7 KO cerebral cortex (Figure 2C). Underlined mechanisms of the upregulation were unknown. Since the downregulation of other Beta3Gn-T family members was not observed in the Beta3Gn-T7 KO brain, we concluded that Beta3Gn-T7 is essential for the synthesis of R-10G-positive KS in the brain of adult mice. Beta3Gn-T7 acts in the pathway of brain KS synthesis in concert with GlcNAc6ST3 and GlcNAc6ST1 (Narentuya et al., 2019; Figure 3). The Beta3Gn-T7 gene was first identified as a gene involved in cell motility (Kataoka and Huh, 2002) and was found to be highly expressed in the intestinal tract, similar to GlcNAc6ST3 (Lee et al., 1999; Lu et al., 2014). The common regulatory mechanism of mRNA expression in oligodendrocytes and intestines is also an interesting topic.

R-10G-positive KS were observed in the perisynaptic regions and in the proximal area of synaptic clefts within dendritic spines (Takeda-Uchimura et al., 2015). The distribution of R-10G-positive KS proteoglycans within the neuropils may be affected in the Beta3Gn-T7 KO brain. This issue will be considered as a future topic. R-10G-positive KS-modified isoforms of Ptprz1/phosphacan may be involved in maintaining the lattice-like PNNs by interacting with NCAM or tenascin (Celio and Chiquet-Ehrismann, 1993; Milev et al., 1994, 1998; Eill et al., 2020) at the close proximity to the neuronal cell surface (Galtrey and Fawcett, 2007). The possibility that oligodendrocyte-derived Beta3Gn-T7, GlcNAc6ST3, and R-10G-positive KS Ptprz1/phosphacan in neuronal plasticity in the adult brain is a further perspective by applying systems of cell-type-specific deletions of *Ptprz1* in Beta3Gn-T7-KO or GlcNAc6ST3-KO mice. Examining the ocular dominance plasticity in Beta3Gn-T7-KO and GlcNAc6ST1 and GlcNAc6ST3 DKO adult mice may address the question of whether the R-10G reactive KS/CS proteoglycan regulates experience-dependent changes in the visual responses of cortical neurons in the adult brain. The ocular dominance shift resulting from monocular deprivation by recording visual evoked potentials from the binocular region of the visual cortex will be assessed in these mice (Takeda-Uchimura et al., 2015). Cat316-positive CS chains occur in the inner PNN, proximal to the surface of the neuronal soma. The molecular relationship between R-10G KS and Cat316-CS is another interesting topic (Nadanaka et al., 2020).

The findings of this study and our previous work clearly indicate that Beat3Gn-T7 is essential for the synthesis of KS in the adult brain. Oligodendrocyte lineage cells are a major source of the R-10G-positive KS. It is likely that Beta3Gn-T7 in OPCs and oligodendrocytes post-translationally modifies phosphacan, which is then translocated into neuropils and the vicinity of a subset of neurons. The possible contribution of oligodendrocyte subsets to the formation of PNNs (Kohnke et al., 2021) and the differentiation of PNN-positive neurons through the secretion of R-10G-positive KS Ptprz1/phosphacan is an important topic. A possible link between the R-10G-positive KS proteoglycan in oligodendrocyte lineage cells and neuronal function could be a subject for future research.

## Data Availability Statement

The raw data supporting the conclusions of this article will be made available by the authors, without undue reservation.

## Ethics Statement

The animal study was reviewed and approved by the Animal Research Committee of the authors’ institutions.

## Author Contributions

YT-U, KN, MI, and KU performed the experiments. TOA, YI, and FA contributed reagents and tools. YT-U, KN, and KU interpreted the data and wrote the manuscript. KU designed the study, supervised the project, and took full responsibility for this manuscript. All authors reviewed the results and approved the final version of the manuscript.

## Conflict of Interest

The authors declare that the research was conducted in the absence of any commercial or financial relationships that could be construed as a potential conflict of interest.

## Publisher’s Note

All claims expressed in this article are solely those of the authors and do not necessarily represent those of their affiliated organizations, or those of the publisher, the editors and the reviewers. Any product that may be evaluated in this article, or claim that may be made by its manufacturer, is not guaranteed or endorsed by the publisher.

## Footnotes

1
http://imagej.nih.gov/ij/

2
http://web.stanford.edu/group/barres_lab/brain_rnaseq.html
